# Lotus leaf-inspired thermal insulation and anti-icing topography

**DOI:** 10.1039/d4ra02843k

**Published:** 2024-06-11

**Authors:** Jianjun Cheng, Yi Zhu, Fei Zhan, Lei Wang

**Affiliations:** a Key Laboratory of Advanced Marine Materials, Ningbo Institute of Materials Technology and Engineering, Chinese Academy of Sciences Ningbo 315201 China; b Beijing Key Laboratory of Lignocellulosic Chemistry, Beijing Forestry University Beijing 100083 China leiwangns@bjfu.edu.cn

## Abstract

Porous sandwich-like structures with surface roughness possess the capacity to sustain droplets, diminish the area of contact between solids and liquids, and augment heat conductivity, and thus delay ice formation when the temperature drops below the freezing point. The prevalence of this combination of surface roughness and a hollow sandwich structure has been observed in several organisms, such as lotus leaves, which have developed these features as a result of environmental adaptation. This study introduces a new design for a surface consisting of a micro–nano conical array and a foam structure with a gradient of pores. The primary components of this design were isocyanate and polyether. The hollow gradient sandwich structure was created by manipulating the water content to increase the porosity, resulting in the formation of a conical–pit morphology on the underside of the specimen. This configuration significantly decreased the amount of heat lost and the modulus of elasticity of the sample. Additionally, the micro–nano hydrophobic structure on the upper surface hindered the transmission of temperature and delayed the formation of ice. This concept, inspired by natural structures, has significant potential applications in the areas of anti-icing, energy conservation, and environmental preservation.

## Abstract 2

The rough surface and porous interlayer of lotus leaf transfers its stress from its upper surface to the interlayer, which is bio-fabricated by integrating a micro-cone array surface and porous interlayer.

## Graphical abstract

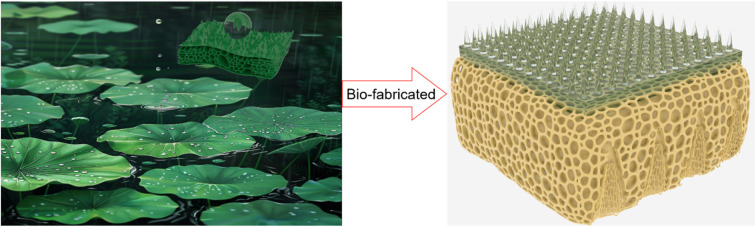

## 1. Introduction

Given the extensive use of fossil fuels globally, there is a growing focus on reducing carbon emissions and protecting the environment. Thermal insulation materials used for building facades can offer some level of thermal insulation. However, their inadequate mechanical strength and lack of resistance to ice formation have raised significant concerns. Thus, a well-designed thermal insulation structure could significantly decrease energy usage and would be a highly promising environmentally friendly material. Numerous biological tissues or organs in nature possess a hollow structure, which serves the purpose of being lightweight and highly durable. Bamboo poles, petioles, and bones exhibit hollow sandwich structures with varying levels of porosity, which significantly enhances their ability to withstand bending and torsion forces.^1–4^ Another important role is the ability to absorb energy and provide thermal insulation. For instance, pomelo peel's structure with varying levels of porosity can efficiently shield the fruit and outer peel by distributing stress and strain.^5–7^ This design, which draws inspiration from the hollow sandwich-like structures seen in nature, is commonly employed in packaging and insulation.^8,9^ In addition, the porous interfacial structure offers a significant quantity of stationary air, resulting in a substantial decrease in heat-transfer efficiency. This structure also enables the surface to be resistant to icing and capable of self-cleaning at low temperatures.^10,11^ Researchers have successfully prevented the formation of ice by keeping fluids with low freezing points, such as silicone oil, in structures that feature pores.^12–16^ Nevertheless, this method necessitates the ongoing infusion of silicone oil to compensate for the depletion of silicone oil that is transported away by surface ice.^17–19^ Hence, it is important to discover an anti-icing configuration that possesses noteworthy mechanical characteristics and long-lasting endurance.

## 2. Results and discussion

### 2.1. Sketch of lotus leaf topography

**Fig. 1:**
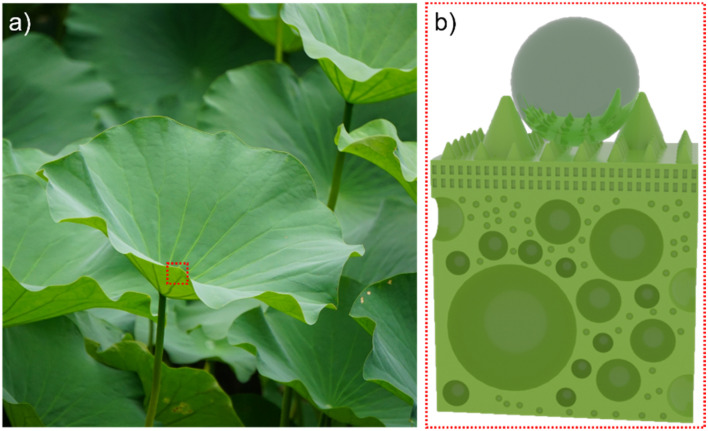
Surface and interlayer structure of the lotus leaf. (a) The lotus leaf in nature that we photographed ourselves. (b) Sketch of lotus leaf surface and interlayer structure.

The lotus leaf stands as the quintessential example of a self-cleaning surface in nature, deriving its outstanding water-repellent function primarily from the intricate multistage micro–nano structure and wax-like material adorning its surface.^20–22^ However, the endurance of this surface functionality comes not only from its flexible multistage structure but also, significantly, from its hollow sandwich configuration, facilitating the transmission of surface stress and strain (Fig. 1). This design minimizes damage to the micro–nano structure on its surface caused by external forces, thus enhancing its stability within natural environments.^23,24^ Integrated with the insulating properties of the hollow sandwich, this combination underscores the potential for robust anti-icing capabilities through the fusion of surface topography and a hollow sandwich design.

### 2.2. Topography, thermal insulation, and simulation

**Fig. 2:**
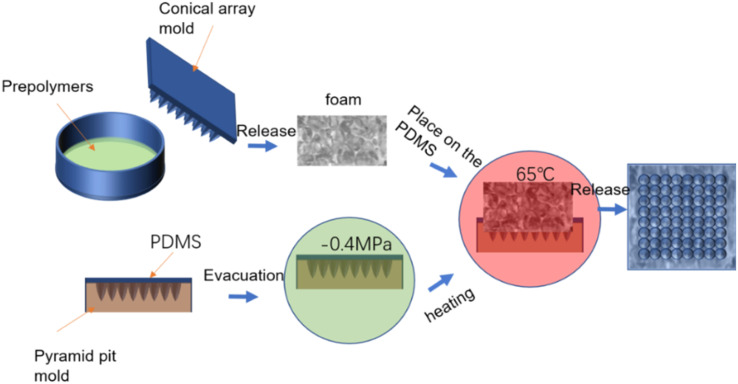
Preparation process of the micro–nano cone array surface and porous gradient sandwich foam structure.

The amalgamation of surface and interface architectures to achieve heightened functionalities is an outcome of natural evolution. For instance, the cooperative influence of lotus leaf's flexible multistage papillae and its honeycomb hollow interlayer facilitates the transmission of stress from the surface to the substrate, thereby mitigating adverse effects on the delicate micro–nano structures atop, consequently enhancing the leaf's structural stability and functional reliability. Such designs are prevalent in nature, warranting further investigation and development. In this study, we propose a foam endowed with a flexible micro/nano-structured surface and a gradient porosity sandwich configuration, affording superhydrophobicity, thermal insulation, and anti-icing capabilities (Fig. 2).

**Fig. 3:**
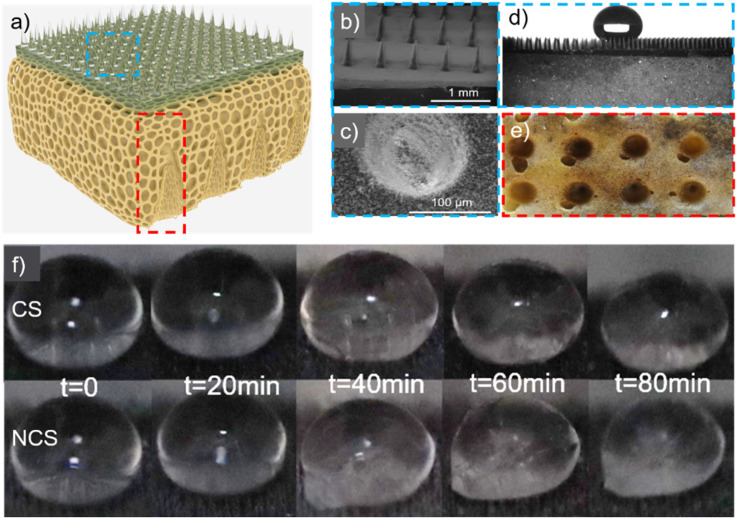
(a) Sketch of the sample topography. (b and c) SEM images of the surface topography. Nano-rods with a length and diameter of 3 μm and 100 nm were observed. (d) Superhydrophobic state of the droplet on the surface. (e) Back details of the sample. (f) Delay in the icing process. The water content of the prepared samples was 1.96%.

In the fabrication of the porous interlayer, a liquid polyurethane foam precursor was selected. The water content critically influences the morphology and mechanical characteristics of the resultant bubbles during foam formation, thereby significantly affecting the foam's flexibility. In this experimental setup, the mechanical properties of the foam were manipulated by adjusting the water content. The introduced water reacted with the isocyanate component of the curing agent, producing carbon dioxide gas. Consequently, upon complete maturation of the foam, a distinct pore gradient was observed within its structure. The low thermal conductivity of air causes thermal insulation of the foam. Finally, the optimal water content of the foam was determined as 1.96% considering the thermal insulation, mechanical properties, and mass. By duplicating the interlayer cross-section, gradient pore interlayers could significantly increase their thermal resistance and surface structural stability, forming a range of conical structures (CSs). The sample without hollow conical–pit arrays was a non-conical structure (NCS). From the bottom to the top surface, the diameter gradually dropped (Fig. 3a).

The sample exhibited noticeable variations in pore size within the foam by the introduction of CO_2_ during the manufacturing process. The droplets on the upper surface exhibited a stable Cassie condition, as depicted in Fig. 3b–d. The contact angle of the upper surface exceeded 150°, indicating the superhydrophobic nature of the upper surface of the sample.^25–27^

The bottom surface of the CS is shown in Fig. 3e. Once the cone-shaped pit structure was created on the lower surface of the sample, the air content within the sample was increased, hence improving the thermal insulation capabilities of the sample. On the other hand, the porosity of the sample was enhanced, leading to a decrease in its elastic modulus and the transfer of stress from the upper surface to the conical position in the interlayer. The CS with small droplets on its upper surface was placed on a cold table (−5 °C) for 30 minutes, whereby the average temperature on the upper surface decreased from 23.8 °C to 22 °C, and the average temperature of the droplets on the upper surface decreased from 18.5 °C to 17.1 °C. The micro–nano structures engineered on the top surface of the foam can encapsulate a substantial volume of air in proximity to water droplets. This configuration resulted in a markedly reduced rate of temperature change in the water droplets compared to that on the unstructured top surface of the foam.

Fig. 3f shows the icing process of droplets on CS and NCS surfaces. The samples were exposed to an environmental temperature of −15 °C, and the state of the droplets was sequentially recorded every 20 minutes. After 40 minutes, the droplet on the NCS upper surface began to freeze and adhere to the sample surface, while the droplet on the CS upper surface did not exhibit freezing. This observation suggested that the design of the CS enhanced the anti-icing capabilities of the sample.

**Fig. 4:**
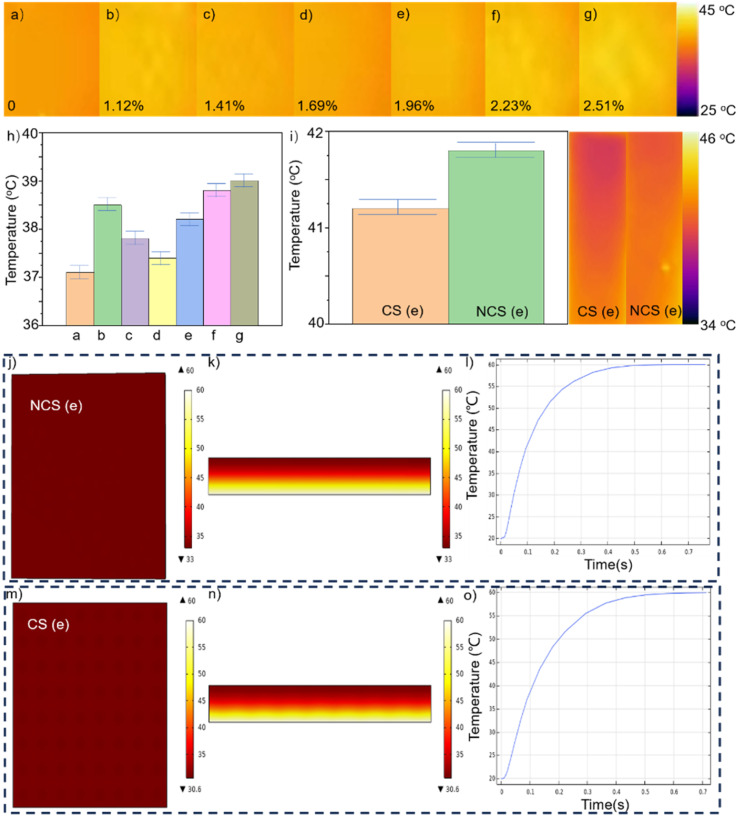
(a–g) Temperature of the upper surface of the sample with water contents of 0%, 1.12%, 1.41%, 1.69%, 1.96%, 2.23%, 2.51% after 20 minutes. (h) Average upper surface temperature of different samples. (i) Comparison of the average temperature of the CS and NCS with a water content of 1.96% (sample) after 25 minutes. Simulation data of NCS (j–l) and CS (m–o).

To evaluate the thermal insulation capabilities, various samples with varying levels of water content were positioned on a preheated heating table set at a temperature of 75 °C. For 20 minutes of heating, Fig. 4 displays the temperature of the upper surface. From Fig. 4a–g, the water content was 0%, 1.12%, 1.41%, 1.69%, 1.96%, 2.23%, and 2.51%, respectively. The foam exhibited optimal thermal insulation efficacy when its water content was completely absent. At a water content of 1.12%, the thermal-insulating capability of the foam experienced a dramatic decline. Once the concentration reached 1.69%, the thermal insulation of the foam achieved a satisfactory level. As the water content increased during the fabrication process, the thermal insulation efficacy of the foam progressively worsened. During the preparation of the foam, the addition of water induced a reaction with the isocyanate to produce carbon dioxide gas. Notably, the thermal conductivity of this gas is considerably low. When the porosity of the foam reached a particular level, further increasing the porosity did not significantly enhance the thermal-insulating capabilities of the foam. However, when the water content increases, the carbon dioxide gas produced will create wider pores within the foam.^28–30^ The foam samples prepared without the addition of water exhibited compact holes internally, with pore sizes smaller than the free path of gas molecules. The motion of gas molecules resulted in collisions with the pore walls, disrupting the heat-transfer process within the gas. Consequently, the thermal conductivity of the gas in the aperture was lower than that of the air. The value of its thermal conductivity is given by the following equation:1
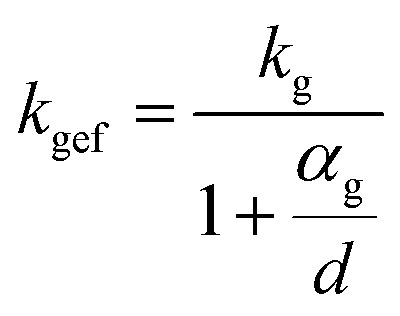
where *k*_g_ is the thermal conductivity of air, *α*_g_ is the free path of gas molecules, and *d* is the pore size.^31,32^

Once the foam is mixed with a little quantity of water, its weight decreases and the porosity of the foam increases. The foam develops larger pores, including pores with an inner diameter exceeding 5 mm. When the inner diameter of the air gap significantly exceeds the mean free path of the gas molecules, the effective thermal conductivity of the air within the gap approaches that of bulk air. Under these conditions, the Knudsen effect becomes negligible. Here, the thermal conductivity of the foam initially increased when the water content reached 0.15%. After adding water, pores with different pore sizes were formed, and the porosity of the foam was greatly improved. The effective thermal conductivity of the air in all the pores of the overall foam is given by the following equations:2
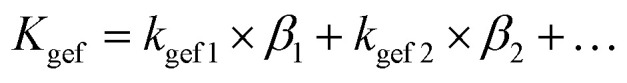

3 *β* = *β*_1_ + *β*_2_ +…

where *k*_gef1_ and *k*_gef2_ represent the effective thermal conductivities of the air within air gaps possessing distinct inner diameters, *β*_1_ and *β*_2_ indicate the corresponding proportions of occupied pores, and *β* is the overall porosity of the foam.^34–39^ The effective thermal conductivity of the solid component of the foam can be determined by the following equation:4
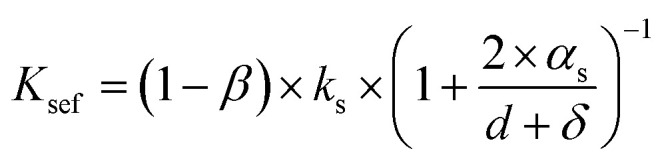
where *α*_s_ is the free path of solid molecules, *k*_s_ is the inherent thermal conductivity of the foam, and *δ* is the thickness of the cell wall. The size parameters, denoted as *d* + *δ*, of the sample are usually greater than 10 nm or even on the micron scale.^40–43^

Increasing the porosity can diminish the role of the foamed solid in the thermal conductivity of the specimen. When porosity was maintained as constant, reducing the pore size amplified the influence of the Knudsen effect, thereby enhancing the thermal insulation performance of the sample. Additionally, when the water content was incrementally increased from 1.12% to 1.96%, the porosity of the foam reached its maximum value. Simultaneously, the pore size of foams exhibited a progressive increase. At a water content of 1.96%, the presence of pores with an inner diameter exceeding 5 mm became evident. The thermal insulation performance of the sample could diminish owing to thermal convection. Consequently, as the water content increased, the thermal insulation performance of the material was more likely to initially deteriorate, subsequently improve, and then deteriorate again. At a water content of 1.69%, the thermal insulation performance was superior to that of other samples including water. Additionally, the average temperature of the upper surface was measured at 37.4 °C. At a water concentration of 1.96%, the upper surface maintained an average temperature of 38.2 °C. Despite a loss in thermal insulation effectiveness, this sample exhibited superior flexibility compared to other samples. Based on the sample with a water content of 1.96%, a hollow conical structure was fabricated at its interlayer. Fig. 4i displays both the mean temperature and the infrared pictures. The mean temperature on the upper surface of NCS (e) registered 41.8 °C, in contrast with 41.2 °C for the upper surface of CS (e). The presence of the cone–pit structure reduced the surface area of the direct contact between the foam floor and the heating table, hence improving the thermal insulation capabilities of the foam. According to the simulation data, the image without the pit structure at the bottom had a minimum temperature of 33 °C, whereas the image with the pit structure at the bottom had a minimum temperature of 30.6 °C. Due to the fixed temperature of 60 °C at the bottom, the highest temperature observed was also 60 °C. The surface of the NCS appeared as a uniformly distributed single color. On the surface of the CS, a relatively darker color was evenly distributed, and these darker areas corresponded to the positions with the conical–pit structure at the bottom. Fig. 4l exhibits a steeper slope compared to Fig. 4o, suggesting that CS provided superior thermal insulation compared to NCS.

### 2.3. Mechanical properties and simulation

**Fig. 5:**
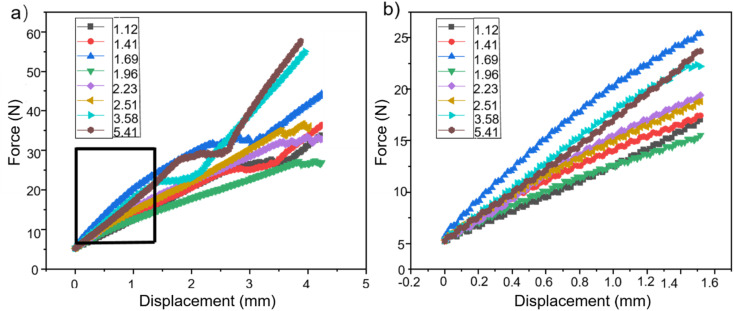
(a) Compression testing under varying water content conditions; (b) localized magnification view of the box area in (a).

During the foaming process, water has a reaction with isocyanate in the foaming agent, resulting in the production of carbon dioxide (CO_2_). This reaction continues until the foaming is fully developed and reaches maturity. Not all of the CO_2_ will be removed from the mixture. The pore structure is formed as a result of the residual CO_2_'s influence on foam production. However, another byproduct of the reaction between water and isocyanate is the formation of a polyurea structure, which promotes chain growth and cross-linking. This, in turn, results in an increase in the brittleness of the foam.^44,45^ The modulus of elasticity and tensile properties of foams can vary depending on the quality of water employed in the foaming solution, as shown in Fig. 5.

**Fig. 6:**
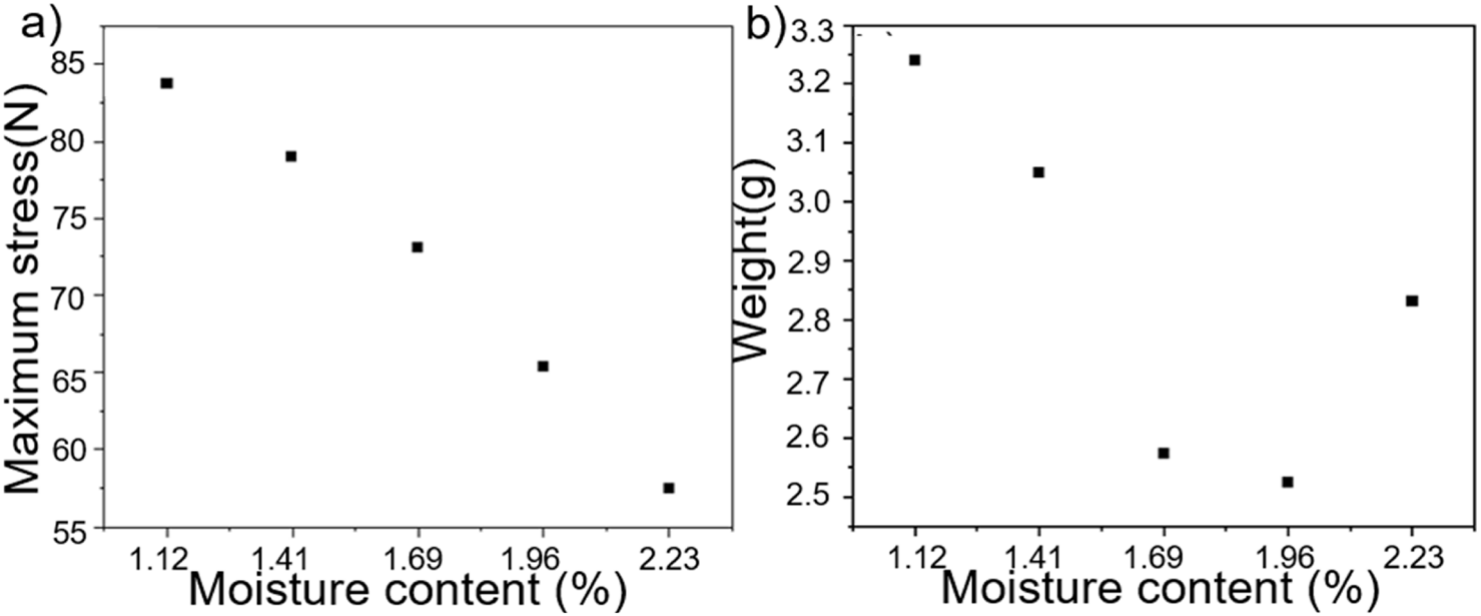
(a) Maximum tensile stress with different water contents. (b) The mass of the same volume with different water contents.

When the compression was large enough, the maximum force value also increased from 33.9 N to 48.8 N as the water content gradually increased from 1.12% to 1.69%. However, when the water content was raised to 1.96%, the foam's maximum stress value was lowered to 27.6 N. Subsequently, as the water volume gradually increased, the foam's maximum force value also increased gradually. When the water content was 5.41%, the maximum force value of the foam was 57.6 N. As the water content increased from 1.12% to 1.69%, the elastic modulus of the foam gradually increased. At a water content of 1.96%, the foam experienced a dramatic reduction in its elastic modulus, which became lower than the elastic modulus at a water content of 1.12%. Subsequently, as the water volume gradually increased, the elastic modulus of the foam started to grow. Fig. 6 displays the tensile characteristics of various samples.

**Fig. 7:**
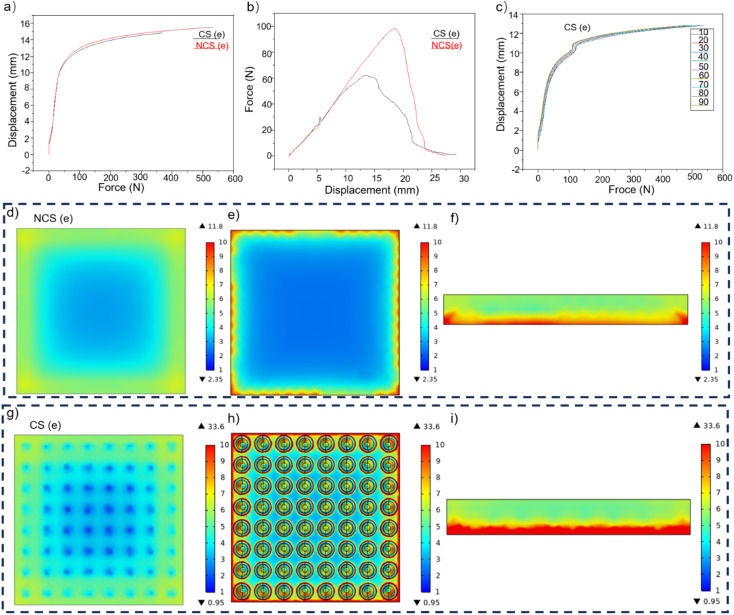
(a) Comparison of the compressive mechanical properties. (b) Comparison of the tensile mechanical properties. (c) Fatigue resistance of repeated compression. (d–f) Top view, bottom view, and side view of the simulation of the structure without cone–pits at the bottom, respectively. (g–i) Simulation of the distribution of the NCS and CS.

At a water content of 1.96%, the maximum tensile force was 65.4 N, and the tensile property diminished linearly as water was added. However, the quality of the product was at its lowest when the water content reached 1.96%, resulting in the highest expansion rate and largest porosity. By utilizing the same quantity of raw material, it is possible to generate a greater volume of foam, hence diminishing manufacturing expenses. A CS foam with a water content of 1.96% was thus developed, taking into account its thermal insulation performance, mechanical performance, and production cost. Fig. 7 displays the comparison of the mechanical properties of the CS and NCS samples.

Fig. 7a demonstrates that the mechanical properties of the sample were minimally impacted by the hollow conical–pit construction at the bottom when the compressive force was below 45 N. When the force exceeded 45 N, both samples experienced equal compressive stress. The sample containing the conical structure exhibited a greater degree of compression, suggesting that the conical structure at the base enhanced the flexibility of the sample. When the top surface of the sample was hit, it experienced more compression and had a longer period to protect the hydrophobic micro–nano structure on the top surface. This protection helped to transfer the stress from the top surface to the pores on the bottom, thus reducing stress on the top surface.^5–7,14–21,28–33,46^Fig. 7b indicates that the sample containing a cone at the bottom could endure a maximum tensile stress of 95 N, whereas the sample lacking the cone structure at the bottom started to fracture once the stress reached 60 N. This suggests that the sample with a cone at the bottom and pit structure at the interlayer had significantly enhanced tensile properties. When the sample underwent 90 consecutive compressions, its compression curve remained mostly unchanged, as shown in Fig. 7c. This suggests that the sample maintained its outstanding mechanical properties even after suffering numerous severe hits. In addition, the pores inside the foam and the conical–pit structure on the ground were beneficial to reduce the elastic modulus of the foam. Given the same level of porosity, pores with larger pore size can undergo significant deformation when subjected to a smaller static load, whereas pores with a smaller pore size can also experience significant deformation. Greater magnitudes of force are necessary to generate lesser amounts of distortion, hence, when considering the same levels of permeability, larger cavities are more advantageous in diminishing the foam's elastic modulus. The correlation between the elastic modulus of the foam and the porosity is given by the following equation:

5 *E* = *E*_0_^(−*βb*)^

where *E*_0_ is the intrinsic elastic modulus of the foam, *β* is the porosity of the foam, and *b* is the material constant of the foam.^46–49^

The simulation results indicated that the stress on the upper surface of the CS model is considerably lower than that of the NCS model at the bottom. This difference is primarily observed at the location corresponding to the cone–pit structure within the interlayer. The tensile tension on the upper surface of the NCS is greater than that on the CS. Upon the addition of the cone structure at the bottom, the maximum stress of the model experienced a rise from 11.8 to 33.6 Pa, while the minimum stress underwent a decrease from 2.35 to 0.95 Pa. The mean stress on the upper surface dropped from 4.5535 to 4.4895 Pa, while the compression magnitude increased from 2.5178 × 10^−5^ to 3.1999 × 10^−5^ mm. The lower cone structure serves to distribute stress from the upper surface to the bottom, so alleviating tension on the upper surface and increasing stress on the lower surface. This helps to safeguard the micro–nano hydrophobic structure on the upper surface. Presently, the foam serves as the main structure, the micro-cone array on the PDMS acts as a secondary structure, and the micro–nano structure on the micro cone functions as a tertiary structure. The presence of a hierarchical structure distribution on the foam surface serves to decrease the elastic modulus of the sample and shield the delicate hydrophobic micro–nano structure on the upper surface.^50–54^

## 3. Conclusion

This work was motivated by examination of the surface and interlayer morphology characteristic of natural lotus leaves. A foam substrate featuring a gradient porous sandwich structure was engineered through meticulous control of the water content. The upper surface of this substrate was subsequently modified to incorporate micro-conical arrays utilizing a reverse molding technique. Both experimental assessments and computational simulations were conducted, which collectively confirmed that the developed foam manifested exceptional anti-icing capabilities and thermal insulation properties. Furthermore, the material demonstrated substantial mechanical resilience, enduring up to 100 compressive cycles, while maintaining low production costs. In conclusion, this innovative design not only facilitates scalability but also heralds a novel methodology for the amalgamation of diverse material properties, particularly in the domains of anti-icing and thermal insulation.

## 4. Experimental section

### 4.1. Preparation of flexible samples

First, 2.5 g of foaming agent (Beijing Haibei Si Technology Co., Ltd), 1 g of curing agent, and 0.07 g of water were added to a disposable Petri dish, stirred quickly and evenly, covered and pressed into an iron mold with a cone array on the upper side, the diameter of which was 5 mm and the height 10 mm. This was then aged in a vacuum drying oven at 65 °C. Next, 40 g polydimethylsiloxane (PDMS) was poured in a mold with a cone pit, wherein the diameter of the cone pit was 0.5 mm, the height was 2 mm, and the mass ratio of PDMS to curing agent was 10 : 1. The mold was put into a vacuum drying box at −0.4 MPa condition and subjected to vacuum treatment for 20 minutes. Afterward, the mold was taken out, placed in the bottom side of the prepared polyurethane foam on the uncured PDMS, and then placed in an oven at 65 °C for heating for 3 h. After the PDMS was solidified, it combined with the foam. Combined together, the final sample was a micro-cone array on PDMS, and a polyurethane foam with a cone–pit array at the interlayer. The preparation process is shown in Fig. 2.

### 4.2. Fabrication of nano-rods

For the formation of nanostructures on its upper surface with a micro-cone structure, graphene was uniformly coated on the surface, and then 0.35 g of hexamethylenetetramine was added to 100 ml of deionized water, and stirred for 3 minutes to completely dissolve it in deionized water. To ionized water was then added 0.74 g of zinc nitrate, and stirred for 5 min to dissolve it completely. The prepared solution was then placed in the reactor, and the sample was put into the solution in the reactor, and the reactor was placed in a vacuum drying oven at 95 °C for 12 h to dry the sample after the reaction. Afterwards, 3 ml of heptadeca fluorodecyltri-propoxysilane was added into the vacuum desiccator, and then the sample was put into the vacuum desiccator, which was evacuated to −0.04 MPa, and then dried in a vacuum. The device was placed in an empty drying box and reacted at 95 °C for 8 h. The obtained sample had excellent hydrophobic properties.

### 4.3. Measurement and characterization

#### 4.3.1. Scanning electron microscopy

The SEM images of the samples were observed by environmental scanning electron microscopy (ESEM, Quanta FEG 250, FEI) under 10 kV voltage and a low vacuum of 900 Pa.

#### 4.3.2. Mechanical property characterization

In this experiment, the mechanical properties of the samples were tested using a Shimadzu AGS-X Tester at a loading rate of 5 mm min^−1^.

#### 4.3.3. Mechanical simulation

The stress-deformation distribution of the topography was simulated using the commercial software COMSOL. In the simulated system, the Young's modulus was 27 778 Pa, the Poisson's ratio was 0.32, and the density of foam was 40 kg m^−3^. The pressure load was 0.01 N. In the calculation process, the compressive load was applied instantaneously, and after it reached a stable state, the relationship between the applied load and the compressive displacement was recorded to obtain the compressive properties of the material.

#### 4.3.4. Icing delay test

A droplet of the same volume of 50 μL was placed on the upper surfaces of the CS and NCS. The samples were placed in a low temperature environment of −15 °C, and the droplet state was recorded every 20 min until the droplet on one of the samples was obviously frozen.

#### 4.3.5. Initial conditions of the thermal-transfer simulation

In setting the initial conditions of the heat-transfer simulation, the material was set as copper to make the results more obvious, the initial temperature of the material itself was 25 °C, the bottom heating temperature was 60 °C, and the surrounding vertical walls and the horizontal walls on the upper surface and the outside air were naturally convective for heat transfer.

## Data availability

The data that support the findings of this study are available from the corresponding author upon reasonable request.

## Author contributions

Lei Wang supervised the research and designed the experiment, Jianjun Cheng, Yi Zhu and Fei Zhan wrote the paper, Fei Zhan simulated the experimental data.

## Conflicts of interest

There are no conflicts to declare.

## Supplementary Material
